# Metallization-Induced Quantum Limits of Contact Resistance in Graphene Nanoribbons with One-Dimensional Contacts

**DOI:** 10.3390/ma14133670

**Published:** 2021-06-30

**Authors:** Mirko Poljak, Mislav Matić

**Affiliations:** Computational Nanoelectronics Group, Faculty of Electrical Engineering and Computing, University of Zagreb, HR 10000 Zagreb, Croatia; mislav.matic@fer.hr

**Keywords:** graphene nanoribbon, contact resistance, edge contact, one-dimensional contact, metallization, quantum transport, NEGF

## Abstract

Graphene has attracted a lot of interest as a potential replacement for silicon in future integrated circuits due to its remarkable electronic and transport properties. In order to meet technology requirements for an acceptable bandgap, graphene needs to be patterned into graphene nanoribbons (GNRs), while one-dimensional (1D) edge metal contacts (MCs) are needed to allow for the encapsulation and preservation of the transport properties. While the properties of GNRs with ideal contacts have been studied extensively, little is known about the electronic and transport properties of GNRs with 1D edge MCs, including contact resistance (*R_C_*), which is one of the key device parameters. In this work, we employ atomistic quantum transport simulations of GNRs with MCs modeled with the wide-band limit (WBL) approach to explore their metallization effects and contact resistance. By studying density of states (DOS), transmission and conductance, we find that metallization decreases transmission and conductance, and either enlarges or diminishes the transport gap depending on GNR dimensions. We calculate the intrinsic quantum limit of width-normalized *R_C_* and find that the limit depends on GNR dimensions, decreasing with width downscaling to ~21 Ω∙µm in 0.4 nm-wide GNRs, and increasing with length downscaling up to ~196 Ω∙µm in 5 nm-long GNRs. We demonstrate that 1D edge contacts and size engineering can be used to tune the *R_C_* in GNRs to values lower than those of graphene.

## 1. Introduction

The main contemporary nanoscale silicon transistor, the fin field-effect transistor (FinFET), is already downscaled to channel thickness of a few nanometers with gate lengths scaled under 20 nm. At this scale, silicon devices suffer from strong short-channel and quantum mechanical effects such as reduced electrostatic control and tunneling, respectively, which considerably deteriorate transistor switching between the ON and OFF states. While different technology options are being explored to replace silicon FinFETs, such as nanowire FETs based on germanium and III–V semiconductor materials [1,2], the emergence of two-dimensional (2D) materials has instigated a tremendous amount of interest in exploring their applicability for future electronic devices [3]. The main advantages they bring are perfect electrostatic channel control due to atomic-level thickness combined with excellent electronic and transport properties, leading to potentially high driving currents. Graphene exhibits high mobilities even on oxide substrates [4,5,6], but has no bandgap, which makes it promising for energy conversion and storage technologies [7,8], but unsuitable for digital logic applications. On the other hand, the bandgaps of transition metal dichalcogenides (TMDs) such as MoS_2_ are appropriate for digital switching, but suffer from low carrier mobilities, which limits the ON state performance of MoS_2_ FETs [9]. Therefore, the search for the 2D material that could replace silicon in ultra-scaled Si devices continues, with recent experimental efforts reported for phosphorene [10,11], silicene [12,13], germanene [14,15], arsenene [16], antimonene [17], and others. Concerning graphene, most studies were focused on large-area graphene devices, but graphene nanostructures such as graphene nanoribbons (GNRs) are needed for ultra-scaled metal-oxide-semiconductor field-effect transistors (MOSFETs), in order to open a bandgap and to meet the demands of the complementary MOS (CMOS) technology for high-density integration on the chips [18,19,20].

Currently, the most severe limiter on 2D material-based device performance is the contact resistance (*R_C_*), which ranges from 100 s to 1000 s Ω∙µm [21,22,23,24]. Its detrimental impact is visible in the reduction of the current driving capabilities since part of the applied bias is lost on the parasitic resistance. In terms of contact configuration for ultra-scaled 2D material MOSFETs, one-dimensional (1D) edge-contacts seem especially promising since they are scalable, unlike conventional top-contacts that are limited by current transfer length, and because they allow for the encapsulation of the 2D material that preserves its electronic and transport properties [25,26]. The lowest reported *R_C_* of graphene devices is under 100 Ω∙µm [22,27], but while these *R_C_* levels are acceptable for future logic technology generations where *R_C_* < 135 Ω∙µm will be needed according to the International Roadmap for Devices and Systems (IRDS) [28], they are still much higher than the elusive quantum limit of contact resistance, which is estimated to be ~30 Ω∙µm [29] to ~90 Ω∙µm [30] for large-area graphene. We have previously studied the influence of series resistance on the performance of 2D material nanoribbon MOSFETs by adding it phenomenologically into the device characteristics in post-processing [31,32]. Moreover, other extrinsic effects such as the existence of crystal defects and their impact on the material and device properties have been studied previously for graphene and other 2D material nanoribbons [33,34,35,36,37,38]. However, there is a clear knowledge gap about the contact resistance in GNR devices with 1D edge-contacts. The device community is especially interested in intrinsic limits to *R_C_*, stemming from the quantum-mechanical electron transport across the metal-GNR interfaces, because the lower limits to *R_C_* set the upper limits to GNR device performance.

In this paper, we employ atomistic quantum transport based on a non-equilibrium Green’s function (NEGF) formalism to study the impact on electronic and transport properties of attaching 1D metallic edge contacts to GNRs. Virtually all theoretical and numerical research on 2D material nanoribbon-based MOSFETs assumes ideal contacts in which the crystal and band structures are identical to those of the channel material [39,40,41]. That approach provides upper limits to device performance and, unfortunately, completely ignores the parasitic resistance formed at contact-channel interfaces. Important work has been done previously on analyzing e.g., metal-graphene [42] and metal-phosphorene contacts [43,44] in the top-contact configuration in terms of the effects of metallization on the transmission and density of states. Our work focuses on metallization effects in ultra-scaled GNRs of interest to CMOS technology, with nanoribbon lengths under ~15 nm and widths under ~4 nm. Amongst other results, we find the lower limits of acceptable GNR dimensions that are set by the contact-induced closure of the transport gap. Most importantly, we use NEGF simulations to explore the impact of metal contacts on GNR conductance, which reveals intrinsic quantum limits to contact resistance in GNRs with 1D edge contacts.

## 2. Materials and Methods

### 2.1. Tight-Binding Hamiltonian

**Figure 1:**
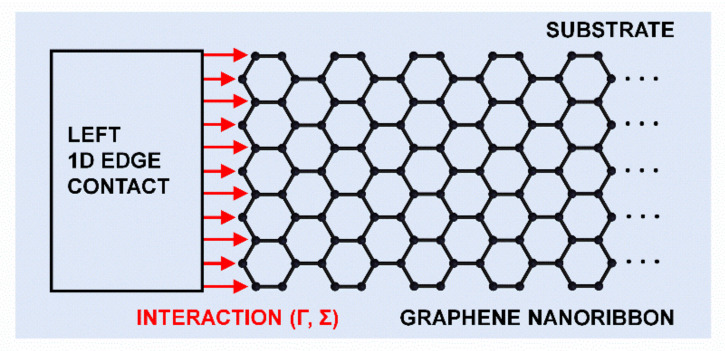
Illustration of the system under study. A graphene nanoribbon is attached to a 1D edge contact that is described with broadening and self-energy within the WBL approximation.

Nanoribbon Hamiltonians are expressed in a multi-band tight-binding (TB) basis, resulting in a full band structure with atomistic resolution. For GNRs, we use a single-orbital TB Hamiltonian (*H*) with the three nearest neighbor interactions included in the model, which is given by.

(1) $$H=\sum_{i}\varepsilon_{i}^{}c_{i}^{†}c_{i}^{}+\sum_{k=1}^{3}t_{k}\sum_{i,j}c_{i}^{†}c_{j}^{}+H.c.,$$

where *ε_i_* is the on-site energy and *c_i_*^†^ (*c_i_*) is the creation (annihilation) operator, while *t*_1_, *t*_2_ and *t*_3_ are the hopping parameters for the nearest, second-nearest, and third-nearest neighbor interactions, including the edge-bond relaxation effect via the modified hopping parameter for edge bonds [19,45]. The total Hamiltonian of the GNR illustrated in Figure 1 in the matrix form is constructed by identifying a 4-atom unit cell, the interaction matrices between unit cells within the same supercell along the GNR width, and the interaction matrices between neighboring supercells [46,47].

### 2.2. Quantum Transport

Geometry-dependent material properties such as the transmission function and density of states (DOS) are obtained by atomistic quantum transport simulations based on the NEGF formalism. The NEGF approach is a state-of-the-art formalism that treats carrier transport fully quantum-mechanically, and accounts for open boundary conditions (OBCs). Including the OBCs is a necessity for all device simulation studies because devices are connected to the outside world via electrical contacts. Moreover, NEGF allows the import of device Hamiltonians of an almost arbitrary complexity, from ab initio to tight-binding ones, which enable the description of the nanodevice under study in sufficient physical detail.

We employ our existing in-house NEGF code previously demonstrated for the analysis of graphene, silicene, germanene, and phosphorene nanostructures [34,37,48]. Within NEGF, the device retarded Green’s function is obtained from.

(2) $$G^{R}(E)={\left[\left(E+i0^{+}\right)I-H-\Sigma_{1}^{R}(E)-\Sigma_{2}^{R}(E)\right]}^{-1},$$

where *E* is the energy, *I* is an identity matrix, *H* is the total device Hamiltonian defined in an identity matrix, *H* is the total device Hamiltonian defined in Section 2.1, and Σ matrices designate the retarded contact self-energies that account for OBCs to the two contacts (left contact or contact 1, and right contact or contact 2). The density of states is found from the spectral function, according to the expression

(3) $$DOS(E)=\frac{2}{2\pi }\mathrm{Trace}\left[i\left(G^{R}(E)-G^{A}(E)\right)\right],$$

where 2 in the numerator is for spin, and *G^A^* is the advanced Green’s function of the device, obtained as (*G^R^*)^†^. The transmission function between the two contacts is calculated using.

(4) $$T(E)=\mathrm{Trace}\left[\Gamma_{1}(E)G^{R}(E)\Gamma_{2}(E)G^{A}(E)\right],$$

where Γ_1,2_ are the contact broadening functions defined as Γ_1,2_ = i (Σ_1,2_ − Σ_1,2_^†^). The conductance of the nanoribbon at *T* = 300 K is calculated using

(5) $$G=G_{0}\int_{0}^{\infty }T(E)\left(-\partial f(E-E_{F})/\partial E\right)dE,$$

where *f*(*E* − *E_F_*) is the Fermi–Dirac function, *E_F_* is the Fermi level, and *G*_0_ = 2 *e*^2^/*h* where 2 in the numerator is for spin and *h* is Planck’s constant. The conductance parameter we focus on is the ON-state conductance (*G_ON_*), evaluated for a Fermi level pushed by 50 meV away from the conduction band minimum (CBM), into the conduction band.

### 2.3. One-Dimensional Contacts

Regarding the two MCs attached to the nanoribbon, we investigate and compare two cases, ideal and metal contacts, but in both cases 1D edge contacts are formed with the nanoribbon, as illustrated in Figure 1.

First, ideal contacts (ICs) assume that contact regions are semi-infinite semiconducting nanoribbons of the same width as the central nanoribbon device that is under study. In this case, surface Green’s functions (SGFs) are found by the computationally efficient Sancho-Rubio method [49]. This configuration is designated as ideal because the central device and contact regions have identical crystal and electronic band structure, which results in a unitary transmission probability for each conducting mode of the GNR.

Second, metal contacts (MCs) are modeled using the wide-band limit (WBL) approach, usually employed in time-dependent quantum transport studies [50], where only the imaginary part of the contact self-energy is retained. The WBL approach is equivalent to assuming a constant DOS at the Fermi level in the contact material and a constant coupling from the metal contact to the channel region. The coupling or interaction between the metal contact and GNR, represented by Γ and Σ, is illustrated in Figure 1. For MC simulations in this work, we set the contact–channel coupling strength to *t* = 3 eV (close to the maximum Slater–Koster parameters in the GNR TB model), and the metal DOS at the Fermi level of 0.2 eV^−1^. While we do not assume any specific metallic material and while we ignore possible Schottky barriers, the chosen metal DOS value corresponds to that of Au (111) contacts connected to a carbon nanotube [51]. Using the WBL approximation replaces iterative procedures for calculating the SGFs, and the two aforementioned parameters lead to constant imaginary elements in the self-energy matrices of metal contacts, i.e., $\Sigma_{1,2}^{R}\approx -i\Gamma /2=-it^{2}g(E_{F})/2$ [52], which gives −ImΣ1,2R=0.9 eV. As illustrated in Figure 1 for the left contact, the metal electrode is connected only to the edge carbon atoms, which implements a 1D edge –contact geometry. This configuration means that the self-energy matrices in Equation (2) will have non-zero entries only for the elements corresponding to or interacting with the edge carbon atoms.

## 3. Results and Discussions

We investigated the impact of attaching WBL MCs on the electronic and transport properties of GNRs such as DOS, transmission, bandgap (*E_G_*) and transport gap (*E_TG_*), conductance, and *R_C_* for GNRs of various lengths (*L*) and widths (*W*) of interest for the potential CMOS technology based on ultra-scaled GNR MOSFETs. In order to meet IRDS requirements on channel length scaling [28], the lengths were under ~15 nm, whereas the widths were under ~4 nm to achieve the appropriate bandgaps for digital switching [18,53].

**Figure 2:**
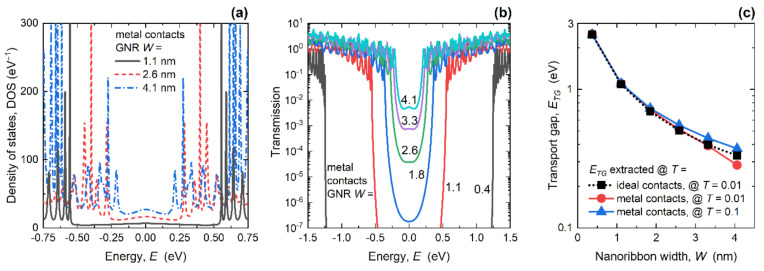
(**a**) Density of states in GNRs with MCs, for various widths; (**b**) impact of width scaling on the transmission of GNRs with MCs; (**c**) band and transport gaps in GNRs with ICs and MCs, respectively. In all cases, *L* = 15.2 nm.

First, we set a common GNR length of ~15 nm and explored metallization effects for various GNR widths. As shown in Figure 2a, attaching 1D MCs induces Lorentzians in the DOS, in contrast to the Van Hove singularities that are observed in GNRs with ideal semi-infinite contacts [42]. Another prominent metallization effect is the occurrence of states inside the bandgap. The metallization-induced DOS increase inside the bandgap is stronger for wider GNRs due to the longer 1D contacts that supply more localized states to the total DOS. As will be shown later, these states are localized in GNR regions that are close to the edge contacts. Consequently, the transport gap still exists, which is fortunate for GNR device applications as the existence of a gap is a necessary condition for digital logic operation.

The existence of a transport gap is visible in the transmission curves reported in Figure 2b for various GNR widths. Namely, inside the bandgap of each GNR, transmission is heavily suppressed, although it almost reaches ~10^−2^ in the widest GNR (*W* = 4.1 nm). Transmission also exhibits MC-induced Lorentzian oscillations in the conduction and valence bands, similarly to the DOS curves, which is in stark contrast to the step-like transmission in GNRs with ideal contacts. In the IC case, the band structures of the central and contact regions are identical, which leads to a unitary transmission probability for each conducting mode [54,55]. On the other hand, WBL MCs are described with constant broadening and self-energy, which enables destructive interference effects on electron waves travelling from WBL MCs into the central GNR region. Consequently, these interference effects cause the occurrence of the transmission Lorentzians and lead to non-unitary transmission probabilities for most energies in the examined spectrum [55].

The transport gap was extracted as the energy range where the transmission was lower than either 0.01 or 0.1, and is shown in Figure 2c for the ICs and MCs of ~15 nm long GNRs of various widths. For the IC case, *E_TG_* coincides with *E_G_* because the ideal contacts do not introduce any kind of scattering or changes in the transmission function, so both approaches give the same energy gap values. In all cases the *E_TG_* increased as *W* is scaled down, as expected, but there were some differences in the *E_TG_* values in GNRs with metal contacts depending on how the *E_TG_* was extracted, especially for wider GNRs. When *E_TG_* was extracted for *T* < 0.01, *E_TG_* was somewhat lower than *E_G_*, which means that metallization increased transmission inside the bandgap. Regarding device applications, this effect is detrimental since it would increase the conductance and current in GNR devices with MCs under conditions where the device should be in the OFF state. When *E_TG_* was extracted for *T* < 0.1, the *E_TG_* was higher than the *E_G_* for *W* > 2 nm, which indicates that the transmission is suppressed near the CBM. Hence, this decrease should result in the lower conductance and current driving capabilities of GNR MOSFETs with MCs in the ON state. While full device simulations and performance analysis are beyond the scope of the current work, we use the IC–MC differences in GNR conductance for the extraction of contact resistance, which plays one of the central roles in GNR MOSFET performance.

**Figure 3:**
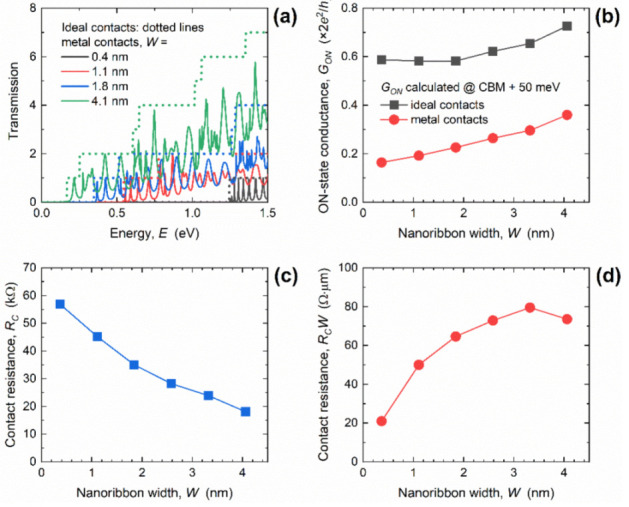
(**a**) Width-dependent transmission in GNRs with ICs and MCs. Impact of GNR width downscaling on (**b**) ON-state conductance, (**c**) contact resistance, and (**d**) width-normalized contact resistance. In all cases, *L* = 15.2 nm.

Figure 3a shows the transmission in the linear scale, which demonstrates step-like characteristics in the case of ICs because GNRs are 1D nanostructures, and oscillatory behavior occurs in the case of WBL MCs attached to the GNR edges. The narrowest GNR shown (*W* = 0.4 nm) exhibited perfect and well-separated Lorentzians in the transmission, whereas the wider nanoribbons show more complicated patterns. These characteristics were due to interference effects coming from the interactions between WBL MCs and a larger number of conducting modes in GNRs of larger widths. Generally, transmission is strongly suppressed over the entire energy range of interest, which should lead to lower conductance in comparison to the IC case.

The impact of *W* downscaling on *G_ON_* for both types of contacts is reported in Figure 3b. The *G_ON_* generally decreases with the decrease of GNR width due to the smaller number of available conduction modes in narrower nanoribbons. While the *G_ON_* in GNRs with ICs decreased from 0.73 to 0.59 *G*_0_, the change went from 0.36 to 0.16 *G*_0_ when *W* was scaled in the GNRs with metal contacts. Attaching MCs decreases *G_ON_* by 51% in 4.1 nm-wide GNRs, and the effect was stronger in narrower nanoribbons where the decrease equals 73% for *W* = 0.4 nm. Since the only difference between the two cases is the description of the contacts, i.e., ideal semi-infinite nanoribbons versus WBL metal contacts with constant broadening, we attribute this conductance decrease to an added contact resistance. Therefore, the additional *R_C_* introduced by WBL MCs is calculated as:

(6) $$R_{C}=\frac{1}{G_{ON(MC)}}-\frac{1}{G_{ON(IC)}},$$

where *G_ON_*_(*MC*)_ and *G_ON_*_(*IC*)_ are the corresponding ON-state conductances for the MC and IC cases, respectively. We stress that Equation (6) provides the added *R_C_* induced by the MCs. Even in the coherent ballistic case with ICs, in which the central device carries no resistance, the intrinsic quantum resistance is formed at contact–device interfaces with a frequently-cited resistance quantum of 12.9 kΩ calculated at 0 K [56].

The dependence of *R_C_* on GNR width for ~15 nm-long GNRs is plotted in Figure 3c. After attaching the MCs, a *R_C_* of 18.1 kΩ was introduced to the contact–GNR interface for *W* = 4.1 nm, while width downscaling increased the resistance to 56.9 kΩ for *W* = 0.4 nm. Narrower GNRs have a smaller number of conducting modes, which results in the observed higher resistance. Attaching 1D edge contacts additionally increases *R_C_* due to the lower transmission in the narrower GNRs with MCs, which was caused by the lower probability of constructive interference (cf. differences between wider and narrower GNRs in Figure 3a). In the device research literature, the *R_C_* is usually normalized by the transistor channel width and expressed as *R_C_W*. Hence, in Figure 3d we plot *R_C_W* in ~15 nm-long GNRs with metal contacts, where *W* is the GNR width. When the width decreased, *R_C_W* also decreased, from 73.5 Ω∙µm in the 4.1 nm-wide GNR down to the surprisingly low 20.9 Ω∙µm in the GNR with *W* = 0.4 nm. The obtained resistance values, as previously stated, represent the added contact resistance introduced by the WBL MCs, so the total *R_C_* visible from the outside world should include the intrinsic interface resistance as well. After including the *G_ON_* results for the IC case from Figure 3b, the total *R_C_W* rises to 29.1 Ω∙µm and 145.9 Ω∙µm for the examined GNR width range. Therefore, lower width-normalized contact resistance was observed in narrower GNRs, which are also more plausible for device applications because an acceptable bandgap is reached for GNR widths under 2.5 nm (see Figure 2c). In comparison to the best reported experimental results for graphene devices with *R_C_* between 84 Ω∙µm and ~100 Ω∙µm [25,57], our results for the GNRs demonstrate that room for improvement exists, since the best measured *R_C_* is at least 2.9× higher than the minimum calculated quantum limit of the total GNR *R_C_*.

**Figure 4:**
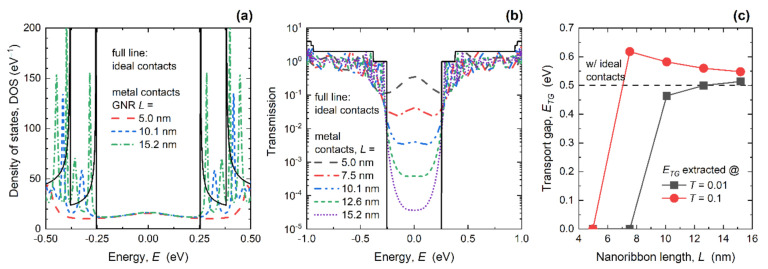
Influence of length downscaling from 15.2 nm to 5.0 nm on (**a**) density of states, (**b**) transmission, and (**c**) the transport gap in GNRs with MCs. In all plots, corresponding results for the IC case are inserted for comparison. *W* = 2.6 nm.

In the next paragraphs, we set a common GNR width of ~2.6 nm and focus on varying the GNR length in the range from ~15 nm down to ~5 nm. This width was chosen as a moderate median value in the examined *W* range, i.e., most parameters saturate for *W* > 4 nm while narrower GNRs exhibit strong width–confinement effects. Channel length scaling has been the main enabler of technology improvement in the semiconductor industry over recent decades and, therefore, we wished to investigate length-dependent metallization effects and contact resistance. As shown in Figure 4a, length scaling leads to qualitatively similar metallization effects in DOS curves as with width scaling, i.e., Van Hove singularities are replaced with broader and shifted Lorentzians. However, the difference is that in the shorter GNRs with MCs, the first Lorentzian peak was much wider and was considerably shifted away from the CBM. The stronger effect in 5 nm-long GNRs occurred as if the MC-induced broadening was effectively increased in shorter nanoribbons. Inside the bandgap, we again observe a non-zero DOS as in Figure 2a, but in this case the number of states did not change with GNR length. This result confirms the previous observation that the mid-gap states are localized, and that the mid-gap DOS quantitatively depends on the GNR width, i.e., number of edge carbon atoms connected to 1D edge contacts.

The impact of decreasing the length on the transmission of the GNRs with MCs is shown in Figure 4b. We observe that, despite the non-zero DOS inside the bandgap, the transport gap was preserved for most GNR lengths. However, for *L* = 5.0 nm and *L* = 7.5 nm the transmission became relatively high, going above 0.01. Clearly, the metallization effects were detrimental for the 5 nm-long GNR because the transport gap closed, which makes them untenable as a channel material for the ultimately scaled GNR MOSFETs. The transport gap behavior detailed in Figure 4c reports the *E_TG_* calculated using the two transmission limits, as was previously done for the *E_TG_* results, in Figure 2c. When the transport gap was defined for *T* < 0.01, the *E_TG_* monotonically decreased and reached zero for *L* ≤ 7.5 nm, due to the MC-induced transmission increase inside the bandgap. In contrast, when *E_TG_* was extracted for *T* < 0.1, the *E_TG_* monotonically increased as *L* decreased due to the strong transmission suppression near the CBM, and then suddenly decreased and reached zero for *L* = 5 nm.

**Figure 5:**
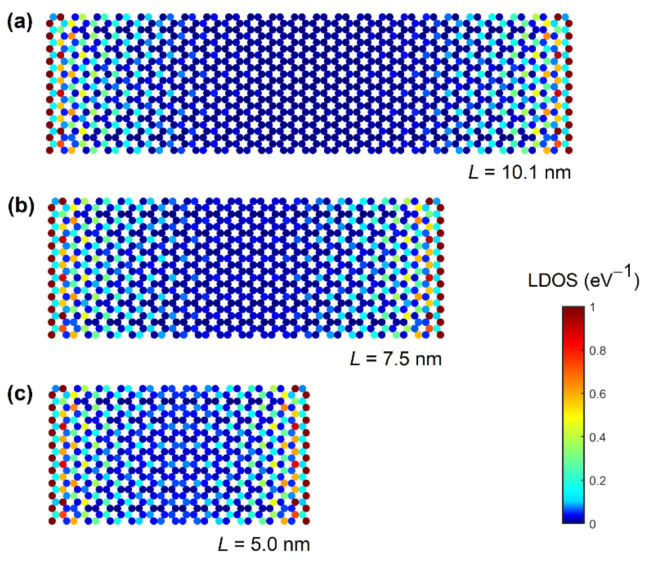
Local density of states in 2.6 nm-wide GNRs with 1D edge contacts for different GNR lengths: (**a**) 10.1 nm, (**b**) 7.5 nm, and (**c**) 5.0 nm.

The increase of transmission within the bandgap in Figure 4b and transport gap closure in Figure 4c can be more readily understood from the atomically-resolved local DOS (LDOS) plots. Figure 5 shows the LDOS at *E* = 0 eV (mid-gap) in ~2.6 nm-wide GNRs of various lengths. When WBL MCs are attached in the edge-contact geometry, we observe increased LDOS in regions close to the contacts. For 10.1 nm-long (Figure 5a) and 7.5 nm-long (Figure 5b) GNRs, these two regions of high LDOS are well separated. In these two cases, carrier transport from one contact to the other was possible only via quantum hopping or tunneling with low probability, which leads to low transmission (see Figure 4b) and non-zero *E_TG_* (see Figure 4c) for the GNRs with the length of 7.5 and 10.1 nm. On the other hand, the two high-LDOS regions touch and interact in the 5 nm-long GNR, as shown in Figure 5c, resulting in an extended non-localized state at 0 eV. Consequently, the extended state through the entire nanoribbon les to a significantly higher transmission probability at *E* = 0 eV than in longer nanoribbons and caused the closing of the transport gap for *L* = 5 nm. Therefore, metallization effects caused by broadening from 1D edge MCs clearly set a lower limit on the acceptable GNR lengths in terms of the transport gap, which reduces the scaling potential of GNRs as a possible channel material in future extremely-scaled CMOS technology.

**Figure 6:**
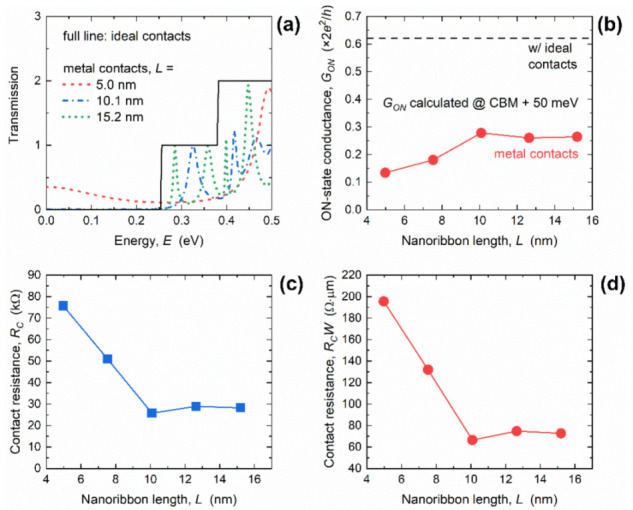
(**a**) Length-dependent transmission in GNRs with ICs and MCs. Impact of length scaling on (**b**) ON-state conductance, (**c**) contact resistance, and (**d**) width-normalized contact resistance. In all cases, *W* = 2.6 nm.

Finally, we turn our attention to the impact of GNR length scaling on conductance and contact resistance in GNRs with MCs. Figure 6a shows the transmission of the 2.6 nm-wide GNR in the linear scale, and again we see a step-like transmission in the case of ICs irrespective of the length, and oscillatory behavior in the case of WBL MCs with severe differences for different lengths. As for the DOS in Figure 4a, length reduction leads to wider and significantly more shifted transmission Lorentzians in shorter GNRs. For the 5 nm-long GNR, the first transmission step occurred at *E* ~ 250 meV with ICs, while the first Lorentzian in GNRs with MCs occurred at *E* ~ 490 meV. When the length increased, transmission Lorentzians shift toward the CBM, as if the broadening and shifting effects were weakening in longer nanoribbons, which was attributed to the larger separation between the two contacts. In addition, transmission increased significantly inside the bandgap for *L* = 5 nm, reaching 0.35 at mid-gap (*E* = 0 eV). All observations indicate the absolute inadequacy of sub-7.5 nm-long GNRs with 1D edge MCs for digital logic applications, due to the expected reduced ON-state and increased OFF-state conductance and, therefore, poor current driving and switching capabilities.

The calculated *G_ON_* is shown in Figure 6b and, while the *G_ON_* was length-independent for the IC case, it generally decreased with length downscaling in GNRs with MCs, from 0.26 *G*_0_ at *L* = 15.2 nm down to 0.13 *G*_0_ for the 5 nm-long GNR. The decrease equals 56% and 79% for the longest and shortest investigated GNRs, respectively. Therefore, the additional *R_C_* introduced by the WBL MCs increases when the length decreases. The *R_C_* was boosted from 28.2 kΩ to 75.8 kΩ when GNR length decreased from 15.2 nm to 5 nm, as reported in Figure 6c. This ~2.7× increase in *R_C_* in the shortest GNRs is associated with strong interaction between the two regions of high LDOS (shown in Figure 5c), and is attributed to the strongly suppressed transmission near the CBM caused by the strong broadening and shift of the first transmission Lorentzian (reported in Figure 6a).

Finally, width-normalized contact resistance (*R_C_W*) is shown in Figure 6d, and it clearly follows an identical trend to the *R_C_*. The *R_C_W* monotonically increased from 72.9 Ω∙µm to 195.7 Ω∙µm in the examined GNR length range. As in the case of width scaling, we include the intrinsic quantum resistance occurring at the contact–nanoribbon interfaces to obtain the total contact resistance in the GNRs with WBL MCs. After including the intrinsic resistance using the the *G_ON_* results for the IC case reported in Figure 6b, the total *R_C_W* rises to 126.6 Ω∙µm for the 15.2 nm-long GNR and 249.4 Ω∙µm for the shortest GNR with *L* = 5 nm. Hence, the worst-case scenario in terms of *R_C_* was obtained for wide and ultra-short GNRs. Nevertheless, 15 nm-long GNRs even in the worst case exhibited contact resistance that was comparable to the quantum limit calculated for large-area graphene [29], which demonstrates that 1D edge contact configuration is promising for ultra-scaled GNR nanodevices.

## 4. Conclusions

Atomistic quantum transport (NEGF) simulations were used to study the electronic and transport properties of GNRs with 1D edge contacts. Employing the WBL approach for moderately-interacting metal electrodes allowed us to explore metallization effects on the transmission and DOS, investigate acceptable GNR dimensions in terms of the conductance and transport gaps, and determine the intrinsic quantum limits of *R_C_* in ultra-scaled GNRs. Generally, metallization effects are significant and are seen in (i) the occurrence of shifted Lorentzian peaks in the transmission and DOS, (ii) the modification of the transport gap, (iii) the considerable reduction of conductance near the CBM and (iv) added contact resistance at the metal–GNR interfaces. Attaching 1D edge contacts sets a lower limit of ~7.5 nm to the acceptable GNR length because metallization effects diminish or completely close the transport gap since MCs induce localized states in GNR regions close to the contacted edges. In shorter GNRs, the separation between these regions decreases and transmission probability inside the bandgap is boosted by the increased interaction. Concerning contact resistance, MCs reduce transmission and conductance, and the decrease is attributed to the added MC-induced *R_C_*. When GNR width decreases, MC-induced *R_C_W* decreases from 73.5 Ω∙µm for the 4.1 nm-wide GNR to 20.9 Ω∙µm for the 0.4 nm-wide GNR. By contrast, MC-induced *R_C_W* increases when GNR length is downscaled, from 72.9 Ω∙µm for the 15.2 nm-long GNR to 195.7 Ω∙µm for the shortest 5 nm-long GNR. We note that the obtained *R_C_* limits for GNRs with *L* > 10 nm are significantly lower than the best reported *R_C_* of 80 Ω∙µm for graphene devices. Therefore, our results indicate that *R_C_* in GNRs with 1D edge contacts can be adjusted by size engineering to levels lower than those of large-area graphene. Moreover, ultra-scaled GNRs could offer competitive contact resistance in line with the requirements set by the electron device research community through IRDS, although care must be taken with length scaling under 7.5 nm due to the metallization-induced decrease of the transport gap. Regarding the fulfilment of IRDS goals, full GNR-based MOSFET device simulations are needed for a complete assessment, which is planned for future work.

## Data Availability

The data presented in this study are contained within the article and are available on request from the corresponding author.
