# Correction: Poljak, M.; Matić, M. Metallization-Induced Quantum Limits of Contact Resistance in Graphene Nanoribbons with One-Dimensional Contacts. *Materials* 2021, *14*, 3670

**DOI:** 10.3390/ma14226965

**Published:** 2021-11-18

**Authors:** Mirko Poljak, Mislav Matić

**Affiliations:** Computational Nanoelectronics Group, Faculty of Electrical Engineering and Computing, University of Zagreb, HR 10000 Zagreb, Croatia; mislav.matic@fer.hr

The authors regret that the results presented in Figure 3c,d and Figure 6c,d in our published paper [1] contain errors. Namely, in the calculations of resistances, we incorrectly used the reduced Planck’s constant instead of Planck’s constant, which led to resistances being lower by a factor of 2π than the correct values. Hereafter, we provide correct versions of Figure 3 and Figure 6.

At several points in the text where the numerical values of *R_C_* and *R_C_W* are mentioned, corrections need to be completed according to the corrected *R_C_* and *R_C_W* data provided in Figure 3 and Figure 6. Nevertheless, the general conclusions of the original paper regarding metallization effects, lower limits of acceptable GNR lengths, qualitative *R_C_* and *R_C_W* behavior, and the main finding that *R_C_* in GNRs with 1D edge contacts can be adjusted by size engineering to levels lower than those of large-area graphene devices, still hold.

The authors would like to apologize for any inconvenience caused to the readers by these changes. The manuscript will be updated, and the original will remain online on the article webpage.

## Figures and Tables

**Figure 3:**
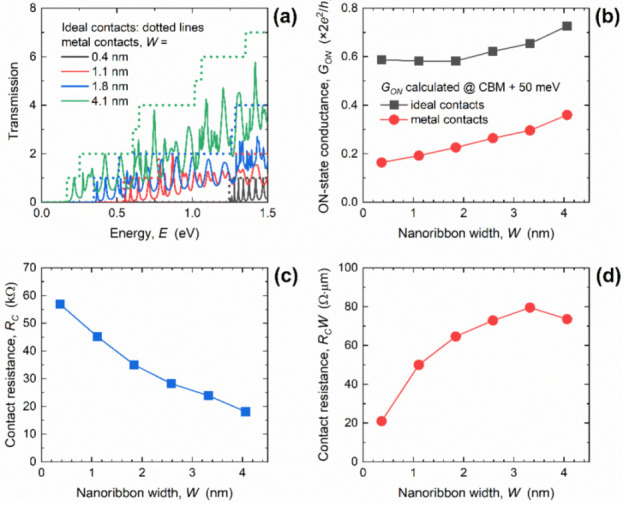
(**a**) Width-dependent transmission in GNRs with ICs and MCs. Impact of GNR width downscaling on (**b**) ON-state conductance, (**c**) contact resistance, and (**d**) width-normalized contact resistance. In all cases, *L* = 15.2 nm.

**Figure 6:**
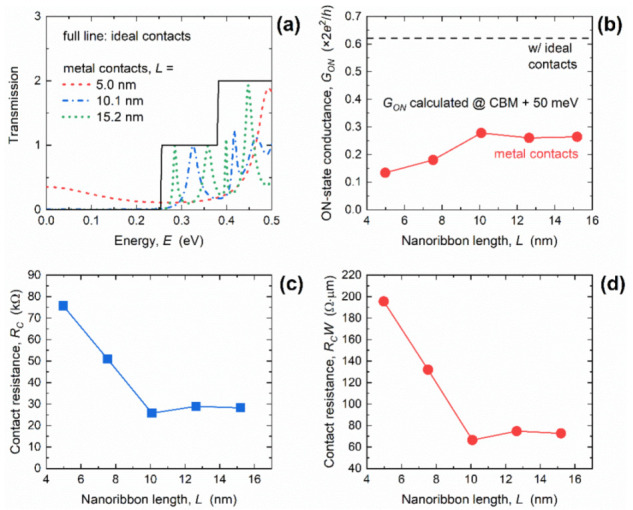
(**a**) Length-dependent transmission in GNRs with ICs and MCs. Impact of length scaling on (**b**) ON-state conductance, (**c**) contact resistance, and (**d**) width-normalized contact resistance. In all cases, *W* = 2.6 nm.
